# Colchicine measurement using LC-MS/MS with ESI in serum with liquid liquid extraction

**DOI:** 10.1186/1546-0096-13-S1-P118

**Published:** 2015-09-28

**Authors:** H Gul, E Demirkaya, B Eser, H Kapucu, V Tuncbilek, D Simsek

**Affiliations:** 1 Gulhane School of Medicine, Ankara, Turkey

## Introduction

Familial Mediterranean fever (FMF) is an inherited autoinflammatory disorder characterized by recurring attacks of fever accompanied by intense pain in the abdomen, chest, or joints. Colchicine, is an important drug which is used in diseases such as FMF and acute gout attacks. However, it has significant side effects such as diarrhea, nausea, vomiting and fatigue. Some of the patients does not respond to treatment due to resistance. Therefore it is important to measure the serum levels of colchicine. The expected serum levels of colchicine is about 5-15 ng/ml. Correct measurement of this level with the classical analytical methods are not easy. Phase extraction and sample concentration of colchicine in routine laboratory conditions is time-consuming, so advanced analytical instruments such as LC-MS/MS and easy sample preparation methods are needed.

## Method

We used Agilent model 6420 LC-MS / MS to measure the serum level of colchicine. Pimozide was used as an internal standard and 100 microliters of IS (7.5 ng/ml) was added into 200 microliters serum. Then serum proteins was precipitated by 900 microliters of methanol and 20 microliters of the supernatant was injected into LC-MS/MS. LC flow rate of 0.5 ml, the phase gradient of 10 mM ammonium acetate + 0.1% formic acid for phase A and the mixture consisted of 0.1% formic acid in methanol for phase B were used.

## Results

Calibration curve for colchicine was constructed with five levels (1.56, 3.1, 6.25, 12.5, 25 ng/ml). r2 value was found to be 0.999. Peak arrival time of 1.9 minutes, the accuracy value of 100.44, the recovery value was found to be 82%. LOD (limit of detection) value was 0.05 ng/ml and the LOQ was 0.1 ng/mL. We tried different substances as an internal standard such as caffeine, cotinine, amlodipine, nifedipine and diltiazem but pimozide was the more stable and consistent, in addition to the least possibility of endogenous existince and reproducibility was found to be the best amongst the others.

## Conclusion

We have developed a method for the measurement of colchicine using LC-MS/MS facilitated the extraction step, and can be applied in routine practice. Dose adjustment may become possible for rational drug use by comparing the serum concentration in response to different doses of colchicine. Thus, to avoid the side effects of medication can be possible with the use of the lowest dose producing responses to the treatment.

